# A review on recent advancements in diagnosis and classification of cancers using artificial intelligence

**DOI:** 10.37796/2211-8039.1012

**Published:** 2020-09-11

**Authors:** Priyanka Ramesh, Ramanathan Karuppasamy, Shanthi Veerappapillai

**Affiliations:** Department of Biotechnology, School of Bio Sciences and Technology, Vellore Institute of Technology, Vellore, 632 014, Tamil Nadu, India

**Keywords:** Artificial intelligence, Artificial neural network, Breast cancer, Gastric cancer, Lung cancer

## Abstract

Artificial intelligence has illustrated drastic changes in radiology and medical imaging techniques which in turn led to tremendous changes in screening patterns. In particular, advancements in these techniques led to the development of computer aided detection (CAD) strategy. These approaches provided highly accurate diagnostic reports which served as a “second-opinion” to the radiologists. However, with significant advancements in artificial intelligence strategy, the diagnostic and classifying capabilities of CAD system are meeting the levels of radiologists and clinicians. Thus, it shifts the CAD system from second opinion approach to a high utility tool. This article reviews the strategies and algorithms developed using artificial intelligence for the foremost cancer diagnosis and classification which overcomes the challenges in the traditional method. In addition, the possible direction of AI in medical aspects is also discussed in this study.

## 1. Introduction

Cancer being one of the non-communicable diseases is ranked at the foremost for being the blockade of survival rate among the global population. For instance 1.8 million new cases and 9.6 million deaths were estimated worldwide in 2018. Among them 59.5% of global cancer deaths had occurred in Asia. Moreover cancer in lung, breast, pancreatic, gastric and skin were the significant contributors for the highest incidence rate in 2018 [1]. Due to the rise of cancer incidence rate, diagnosing of disease using conventional tools at early stage had become difficult. Moreover, these traditional methods experienced diagnostic errors including missed, wrong and delayed cases [2]. On the other hand, under-standing the perplexity of cancer at different stages has complicated the research further. The perplexity of cancer includes early detection, accuracy, tumor evolution, metastasis pattern, recurrence, tumor aggressiveness and determination of tumor margins [3]. To overcome the limitations mentioned above and to diagnose cancer at the earliest, advancement in artificial intelligence (AI) had been raised for quantifying the imaging data.

Deep learning, a section in AI, plays a promising role in automated recognition of features from sample medical images beyond human's role in particular tasks [4]. For instance automated applications of AI enhanced the qualitative potentials of clinicians, which includes tracing numerous lesions at a time, prediction of the resultant tumor by referring to the various databases within a short period, translation of phenotypic variations to genotypic and persistent monitoring of patients. Despite the need for a large quantity of data for training, deep learning had illustrated relative stability against random fluctuations in the ground truth features [5]. Furthermore it generalizes disease imaging techniques with minimal errors which in turn lead to earlier and significant diagnosis of diseases.

**Fig. 1:**
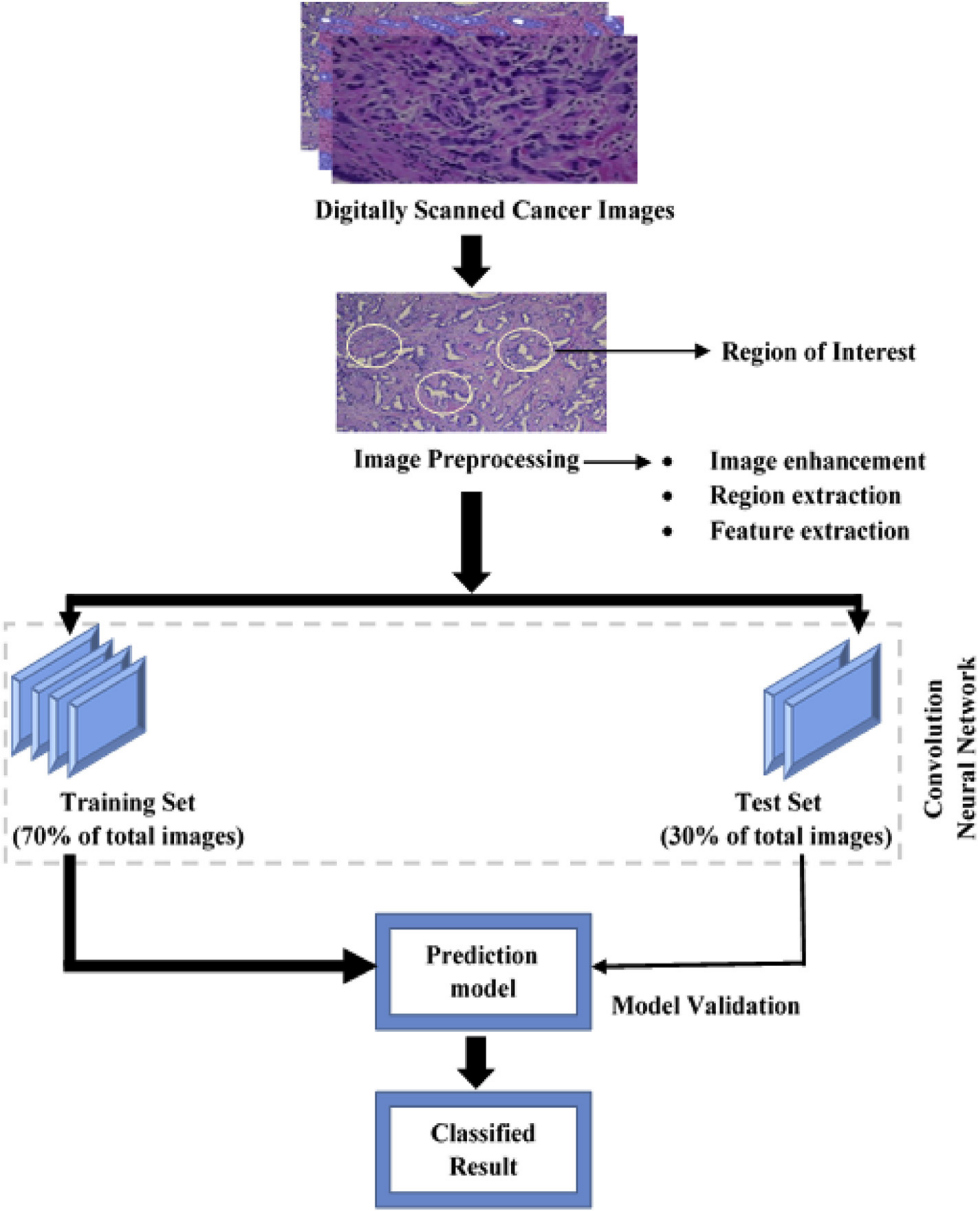
Overall process involved in tumor diagnosis and classification.

Besides deep learning, artificial neural network (ANN) is also used to classify cancer more precisely. It is a mathematical model postulated from the human nervous system comprising of inter-connected neurons. Connectionist strategy is implemented in this technique for computational processing of information. Eventually to illustrate the adaptive system, neural network plays a significant role by modifying its structure based on the training data and by detecting patterns in the data. ANN can be designed through learning process for any particular application [6]. On learning the data during its training time, it organizes itself to proceed further. Moreover many networks can be performed simultaneously and also bears fault tolerance by preventing degradation of structures [7]. Though these studies are intensively applicable in pre-clinical studies, the automated approach of tumor diagnosis and classification discriminates the cancer period of time. The overall process involved in tumor diagnosis and classification were briefed in Fig. 1.

**Fig. 2:**
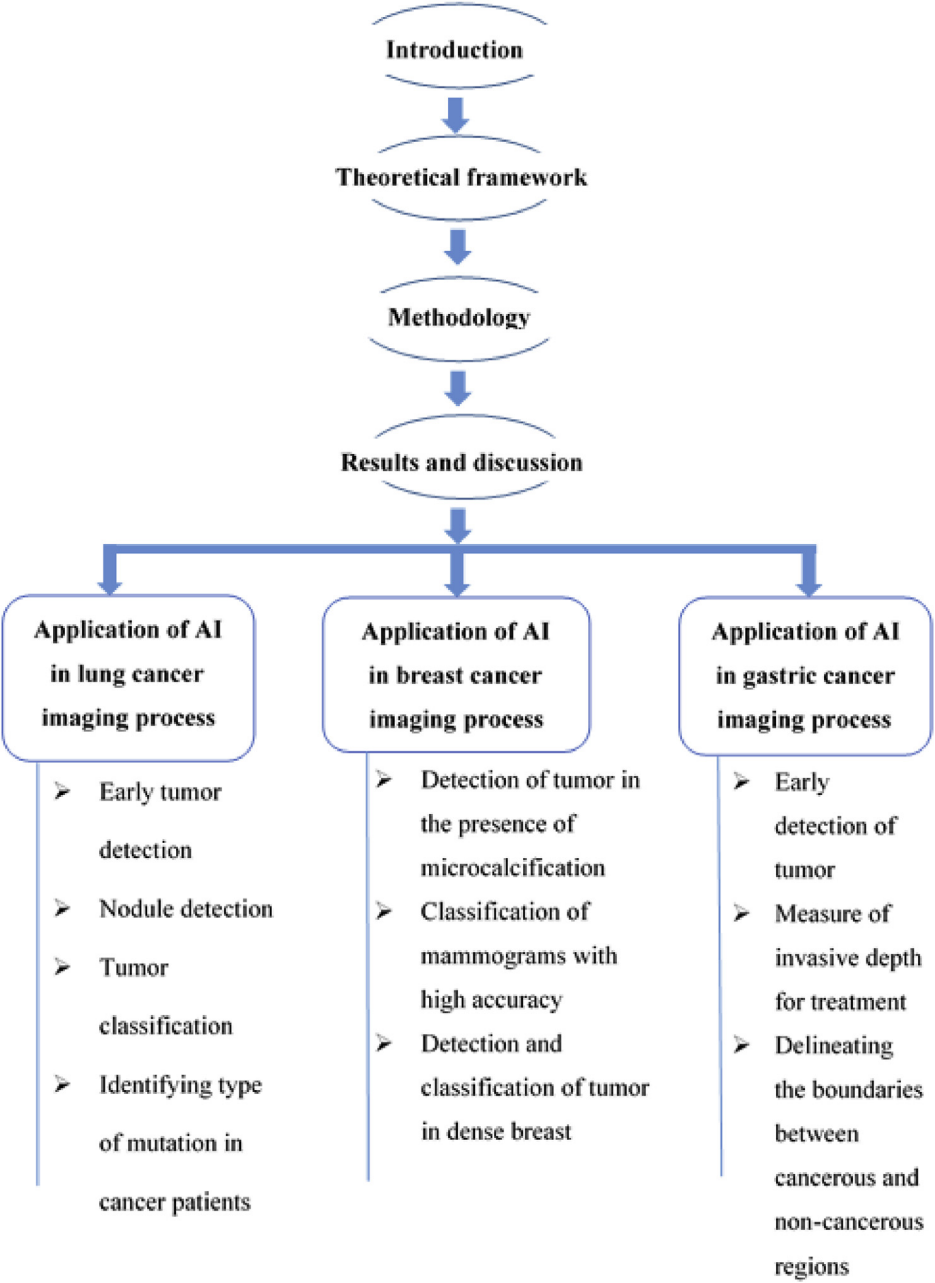
Schematic representation illustrating overall view of our study.

At the light of these evidences, current studies and future applications of AI to medical imaging in top incidental cancers were reviewed during this investigation. This paper highlights the application of AI that generated a drastic transformation in cancer diagnosis and treatment. In particular, the study includes case studies which enlightens application of AI in diagnosis and classification of three cancers (lung, breast and gastric cancer). The evolution of AI in cancer imaging described in this study also provides the significance of AI in cancer diagnosis and treatment. Moreover this study also provides potential applications of AI overcoming the limitations in cancer imaging including presence of denser tissues during diagnosis and classification of cancerous samples from non-tumor samples. The number of case studies concerning AI technology in lung, breast and gastric cancer clarifies the other characteristics of this latest technology. Overall, the study shows the significant application of AI technology which in turn improves the cancer care in the society. Moreover, this paper also suggests that artificial intelligence application in cancer imaging needs further evaluation and validation for improved reliability and generalizability of approaches in clinical practices [8]. The tree diagram provided in Fig. 2 illustrates the structure of the paper for better understanding.

## 2. Theoretical background

AI in oncology imaging process includes three major clinical tasks: Diagnosis, characterization and monitoring. In general, detection involves observation of pathologists over variations in image intensities or complex patterns to understand the aberrations in the patients. Advanced computational technology-assisted pathologists to predict and identify cancer with more accuracy and fewer errors. This strategy of detection is known as Computer Aided Detection (CAD) [9]. AI based tools assisted in identification of missed cancer patients as well as detection with high accuracy and sensitivity [10]. The second task characterization includes diagnosis, segmentation and staging of cancer. This task quantifies abnormality of features including size and texture. Segmentation measures extent of abnormalities in a two dimensional (2D) plane. Manual segmentation process in clinical practice limits itself with minimal accuracy, inter-rater bias, high time and labor consumption and inconsistent reproducibility [11]. In contrast, AI based approach provided automated segmentation with increased quality, reproducibility and efficiency.

Further expansion of technology leads to an integrated method in which separate segmentation process is not required. This task also includes staging of disease in which the cancer is explained based on predefined features and are classified based on TNM classification [12]. Ensemble methods were employed in traditional staging task whereas automated staging relies on tumor size, metastasis and neighboring lymph node data for classification [13].

On the other hand ANN had become a significant strategy for cancer classification. It consists of three consecutive layers namely input layers, hidden layers and output layer. ANN can be classified into single layer feed forward network, single node with its own feedback, multi-layer feed forward network and multi-layer recurrent network. In addition, it also uses three different learning strategy for generating neural network which includes super-vised learning, unsupervised learning and reinforcement learning. Among them supervised learning has higher accuracy and precision as it develops output based on the pattern of each input [7]. Hence automated staging is more reliable than traditional approach. Despite the complexity of data, artificial intelligence is remarkable in identifying the features from large volume of medical data which in turn is used to assist clinicians [14]. It also identifies complicated patterns and supports in transforming images into valuable quantitative information. It is to be noted that these information are not recognized by humans, thus makes clinical decisions easier.

Besides, detection and classification of cancers were the major challenges in the medical field. Artificial intelligence assists clinicians in interpreting cancer images, including cancer stages, tumor delineations, detection of mutations, the impact of anti-cancer treatment, and the influence of disease on other organs. For instance, Khan et al. had pro-posed an integrated convolution neural network algorithm to classify the breast tumor as benign and malignant, which in turn reduced the burden of pathologists in tumor classification [15]. Similarly, a deep learning model developed by Kim et al. and his co-workers assisted the clinicians in predicting the survival rate of the oral cancer patients, which in turn helped the medical practitioners to provide appropriate and effective treatments to the patients [16].

Moreover, medical input data varies beyond imaging techniques, which include blood biomarkers, molecular signatures, and statistical data. It is to be noted that AI is one of the integrative tools that parallel and normalizes various streams of information. Also, advancements in AI approach assists human experts to visualize, understand, and analyze the results [17]. Besides medical imaging, the application of AI in health monitoring enhances efficiency with reduced cost.

Despite radiation dose, subsequent measurements, and scan – time, advancements in phase-contrast imaging techniques can assists radiologists in detecting tumors. This improvement in combination with AI analysis prevents the revisiting of patients with decreased false-positive rates [4,18]. Moreover, advancements in digital pathology will be more quantitative than radiology [19]. With more advancements in AI, we expect designing of FDA approved standard protocols for cancer diagnosis, classification, and management.

## 3. Methodology

In order to manifest the potential of artificial intelligence in cancer imaging, the following case studies were described in this paper:

• Application of artificial intelligence techniques detection and classification of
  ◦ lung cancer
  ◦ Breast cancer
  ◦ Gastric cancer

Furthermore, to show the advancements of AI in the field of cancer, the following search terms were used in the advanced search tool [20]:

• Application of artificial intelligence in lung cancer imaging technique
• Application of artificial intelligence in breast cancer imaging technique
• Application of artificial intelligence in gastric cancer imaging technique

**Fig. 3:**
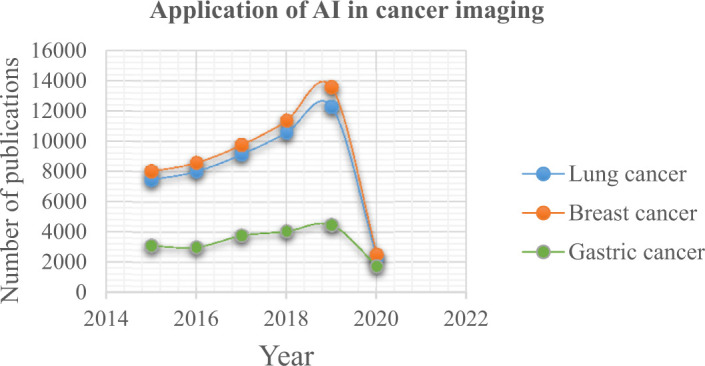
Graph explaining the significance of AI in the field of cancer imaging.

The evolution of artificial intelligence in cancer imaging was carried out for a period of five years (January 2015 to May 2020). In order to understand the significance of AI in cancer, the graphical analysis of the evolution of AI in cancer imaging in the corresponding cancer type is illustrated in Fig. 3. In particular, the recent development of AI in cancer imaging techniques after 2017 is briefly discussed in this study. Selective research works having valuable contributions in the lung, breast and gastric cancer diagnosis and classification is tabulated in Table 1, 2 and 3 respectively.

## 4. Results and Discussion

Recent statistical analysis had reported a drastically increased death rate in lung cancer, breast cancer and gastric cancer worldwide [21]. Hence, early detection and classification with high accuracy had become essential. In turn, the contribution of researchers towards detection and classification had been increased. Fig. 3 shows the significance of AI in cancer imaging by researchers.

### 4.1. Current trends in lung cancer diagnosis and classification using artificial intelligence

Lung adenocarcinoma (LADC) being the foremost cause of death in humans among all other cancers. The morphological features of LADC are heterogeneous in nature, thus provides variation during diagnosis. Depending on the tumor size and lymph location, lung cancer is classified into four stages: stage 1 to stage 4. The survival of victim depends on stages of the cancer which enhances the survival rate of the victim [22]. Hence early diagnosis plays a crucial role in lung cancer.

The preliminary diagnosis of lung cancer depends on the detection of pulmonary nodules. The computed tomography of both benign and malignant tumors contains pulmonary nodules. Hence differentiating those into benign and malignant using visual assessments by the radiologists had become challenging [23]. Moreover, nodule size is the most reliable prognosticator of malignancy in lung cancer. Hence nodule detection had become crucial in early diagnosis.

Till now, the tissue imaging process is playing an essential role in lung cancer prognosis. This strategy depends on morphological features such as tumor size, shape, and invasion of the tumor cells. It is also to be noted that these techniques lack a systematic approach of correlating the features to disease diagnosis [24]. Additionally, other diagnosing systems such as magnetic resonance imaging (MRI), sputum cytology, and chest radiography resulted in poor patient survival due to its lower classification accuracy and higher classification error rate. Hence the application of AI emerged as an effective tool in lung cancer diagnosis and classification.

Recently artificial intelligence is playing a prominent role in tumor detection, segmentation and classification as well as nodule detection in lung cancer. Moreover, tumor classification and metastasis detection from H&E images have been facilitated using deep learning strategies. For instance, Wang et al. and his co-workers developed a shape-based diagnostic model based on features such as age, gender, smoking status, and disease stage. The ultimate aim of the work to characterize the shape of the tumor, which is closely associated with disease prognosis. A convolution neural network was developed using 539 pathology images of lung adenocarcinoma patients obtained from NLST and TCGA repository. The generated model was validated using a subset of 389 images and the tumors were detected. The shape features of the tumor were extracted and analyzed. In addition, the risk score was used to group the people into low risk and high risk respectively. Moreover Kaplan – Meier method was to predict the survival rate of the patients. Around 89.8% accuracy was obtained for the prediction of tumor [25]. This study significantly proves the application of AI in lung cancer diagnosis.

Recently, a combined technique of neural network was proposed by Senthil et al. and his research scholar to enhance the classification accuracy with minimal error rate. They combined particle swarm optimization (PSO) algorithm to the neural network classifier. PSO is a highly cost-effective computational strategy with high speed. The accuracy, specificity and sensitivity of the proposed PSO-neural network (PSO-NN) was compared with the standard neural networks such as k-means neural network (KNN), Bayes network (BN), neural network (NN) and support vector machine (SVM). Accuracy of PSO-NN was about 97.8%, whereas the accuracy of KNN, BN, NN and SVM was found to be 68.9%, 74.6%, 85.4% and 91.5% respectively [26]. The proposed network suggests that AI can be effectively implemented for lung cancer diagnosis. Moreover it assists doctors in diagnosing and medicate patients at the earliest.

Besides the classification of lung cancer patients, the identification of mutations plays an important role in targeted therapy. The deep learning convolutional neural network model built using 1634 histopathology images from TCGA repository assisted the experts in treating patients through targeted therapy. In this study, Nicolas et al. used v3 convolutional neural network to classify disease and to identify the mutations from the images. Using the proposed model, he achieved around 97% of specificity and sensitivity for classifying the samples and 86% accuracy for identifying the mutations from the samples. His findings suggested the experts to cancer types and mutation with high accuracy and less expensive strategy [27]. Thus this proposed work can provide a promising treatment for lung cancer patients. Additionally, some research works contributed to lung cancer diagnosis, and classification were tabulated in Table 1.

### 4.2. Artificial Intelligence in diagnosis and classification of breast cancer

Breast cancer holds the second position amongst overall cancer death worldwide. It occurs due to uncontrolled and abnormal growth of tissues resulting in lump formation in the breast. Thus it consequently leads to tumor growth which can be treated successfully at their early stages [35]. Recently, the most prominent tool “mammography” has been developed for early diagnosis of breast cancer. Regardless mammography had reduced mortality rate by 30%, it contained certain limitations, including high false-positive rates, unnecessary biopsies, overdiagnosis, and treatment [36].

In addition, analyzing of a mammogram differs based on the experience and is always found to be biased due to dense breast tissues [37]. Subsequently, this leads to interval cancers that are detected prior to biennial screening [38].

Similarly, the presence of masses and micro-calcification (calcium deposition) interferes with the quality of mammograms. Moreover, the detection of masses is more challenging than microcalcification due to its variation in size and shape, which in turn produces poor contrast images during mammography. This made the radiologists challenging to classify them as benign and malignant [39]. Thus automated image detection and classification is playing a vital role nowadays.

ANN is one of the most widely used tools for interpreting and decision making of mammography as well as biopsy screenings. The two major applications of AI in breast cancer are feature analysis from the images and to implement classifiers over the desired target [40].

In general, morphology of cells and its components are regulated by biological mechanisms such as differentiation, growth, and development. Earlier pathologists performed tedious visual approaches for tumor grading and morphological assessment of samples. This created larger variations amongst the senior pathologists [41]. Hence many strategies were developed for automated image analysis which includes CAD till AI. Recently, AI based strategies were found to be outperforming in pathology image analysis [42]. For instance, Rakhlin and his research group drawn attention for image feature extraction and their classification. Deep convolution neural network was implemented for extraction of image features, and gradient boosted trees classified them into 2 class as well as 4 class classifications respectively. Their study reported about 93.8% accuracy and 97.3% AUC with respect to 2-class classification tasks, whereas the 4-class classification reported only about 87.2% accuracy. Their strategy implemented unsupervised learning for feature extraction and supervised learning for classification. This study shows a significant result for feature extraction and classification during pathology image analysis [43].

In addition, the presence of highly dense breasts acts as a risk marker during imaging process. It describes the measure of intensity of fibro-glandular tissue in the breast. Moreover presence of dense breast masks cancer and thus reduces the sensitivity during imaging. Besides, the manual classification of dense breast images into four qualitative categories based on Breast Imaging and Reporting Data Systems (BI-RADS) by the radiologists was found to be more difficult [44]. Several studies using deep learning have been investigated in mammography imaging processes. Mohamed et al. and his group investigated a novel Convolution Neural Network (CNN) for classifying mammograms into scattered density and heterogeneously dense samples. The classification performance of CNN was also tested using the refined mammograms during their investigation. The AUC of the CNN model was 94.2%. They observed that increasing the number of mammogram images increased their accuracy from 94.2% to 98.8%. Hence their study demonstrated the classification accuracies between the two groups, which subsequently enhances clinical assessment of breast densities [45].

Despite the advancements in breast imaging techniques, interpreting the patterns have become more challenging. Besides, it required specialization and experience. However, the performance by the radiologists has been depleted due to the high incidence rate of breast cancer. At the same time, increased false-positive and recall rates had criticized the application of mammography for imaging and screening. Regardless of their drawbacks, mammograms have the property of being single-slice projection images which can be trained easily using ANN [46]. Becker et al. illustrated a combinatorial approach of deep learning with CNN (d – CNN) for diagnosing the images with higher accuracy and shorter period of time. They compared the accuracy of d – CNN with the experienced radiologists. The study reported about 82% AUC for the model and about 79% AUC for radiologists respectively. The suggested model for analyzing general mammograms of breast cancer was higher than the radiologists. In a similar manner, the study suggested that improving the models will assist radiologists and makes clinical assessments easier [47]. Further, the contributions of other researchers are tabulated in Table 2.

### 4.3. Recently proposed artificial intelligence strategies in gastric cancer diagnosis and classification

Gastric cancer positions third as a fatal disease and fifth for its high incidence rate in the world [58]. The patients with early gastric cancer rarely bear the symptoms, but later on, the symptoms progress gradually. Moreover, the symptoms are much similar to gastric ulcers, thus it makes it difficult for the patients to differentiate them. This pre-requisites the need for early diagnosis of gastric cancer. The advancement in endoscopy assists in early diagnosis and substantially reduces the mortality rate of gastric cancer. Further, early detection helps us to remove the lesions using endoscopic resection, which significantly improves the patient's health quality [59]. Therefore it is essential to attain early diagnosis with high accuracy for identifying lesions to prevent and treat gastric cancer.

Magnifying endoscopy with narrow band imaging (M-NBI) is used to inspect glandular epithelium by observing microsurface structure and microvascular architecture. It has better accuracy than light endoscopy to distinguish non-cancerous and gastric cancer lesions [60]. Several investigations had re-ported that the sensitivity and specificity of identifying gastric cancer lesions using M-NBI was 85.7-97.3% and 84.4-96.8% respectively [61]. However, the differentiation ability between cancerous and non-cancerous lesions using M-NBI by non-experts was disappointing. To prevail over the limitations above, AI was implemented to enhance the accuracy of medical diagnosis.

Endoscopy is a widely used tool for diagnosing early stage of gastric cancer, among which 7.2% of patients were misdiagnosed. The meager changes in mucosa are often not detected during endoscopic analysis. This requires trained endoscopists with well-armed knowledge [62]. Hence Deep Convolution Neural Network (DCNN) gained attention for predicting and analyzing endoscopic images. Wu et al. detected early gastric cancer lesions with 92.5% accuracy and 94% specificity using DCNN. The diagnosing accuracy and stability were higher than the trained endoscopists. The time taken by DCNN model for diagnosing the lesions was comparatively lower than the endoscopists. Additionally, his study supported the generation of grid model over the stomach which covers the suspicious lesion regions too [63].

DL technology was initially implemented by Hirasawa et al. for the diagnosis of early gastric cancer in the year 2018. Researchers used images from conventional endoscope rather than from FICE and magnifying NBI. Single-shot multi-box pattern was used for the construction of CNN model. About 13,000 images were used as training set and 2296 images as test set for validating the performance of the model. Around 77 images in the test set were found with the lesions. The model categorized the test set in 47 seconds by finding 71 lesions containing images accurately with 92% overall sensitivity of the system. The CNN model detected and processed a stockpile of endoscopic images [64].

Endoscopic submucosal dissection is another approach for treating gastric cancer with minimal invasive depth. This technique is highly preferable by the patients since it requires shorter stay at hospitals [65]. However, endoscopic submucosal dissection are performed to the patients with invasive depth till submucosal layer of stomach based on the guidelines. In addition, the invasion depth can also be determined using endoscopic interventions for early gastric cancer by spraying indigo carmine dye. Moreover, the conventional endoscopy resulted in 69% to 79% accuracy [66]. This shows that there is no reliable tool available for measuring the invasive depth in gastric cancers. Recently CAD has been implemented for differential diagnosis of diseases. In 2018, Zhu et al. deter-mined the invasive extent using CNN – CAD through transfer learning. About 89.16% accuracy was achieved in measuring the invasive depth with 76.47% sensitivity and 95.56% specificity. The developed CNN – CAD model differentiated earlier gastric cancer from submucosal invasion. This study also reduced misconception of invasion depth, which reduces trivial gastrectomy procedures [67]. Other contributions in gastric cancer imaging are consolidated in Table 3.

Another investigation by Kanesaka et al. improved the strategy by developing software to identify and delineate the boundaries between cancerous and non-cancerous regions. Support vector machines were implemented in this study to analyze the gray-level matrix features of narrow-band images. One hundred twenty-six images were used as a training set, and 81 images were used as a test set for constructing the model. The sensitivity and specificity of the model for cancer from non-cancerous imaged were validated and found to be 97% and 95%, respectively. Similarly, the sensitivity and specificity of area concordance were 66% and 81%, respectively [68].

## 5. Conclusion

Artificial intelligence has emerged as a powerful tool in cancer prognosis and management. Advancements in AI can be effectively implemented in personalized treatment and monitoring patient's health leading to a higher quality of care. Other areas in cancer such as follow up of patient's health, biochemical tests require the assistance of AI to help clinicians. Although the application of AI is beneficial, correct steps must be taken for framing the workflow in concern with the medical context to prevent undesired side effects in patients. Over time, this strategy may become a standard approach for human incidental disease diagnosis, evaluation, and reporting of data.

For bioinformaticians, image computing and machine learning assists in discovering prominent features for diagnosis and treatment, overcoming the aforementioned opportunities and challenges. It also assists researchers in developing new algorithms for disease characterization using radiology, molecular, and histology data. In this study, the limitations in the diagnosis and management of cancer diagnosis and classification was addressed. Also, the application of AI in tumor detection and classification using diagnostic images was discussed. A constant increase in the number and complexity of cancer images has reduced the time for evaluating the images by the radiologists. Due to the massive volume of images, prediction of tumors at the earliest has become one of the significant areas to be influenced by artificial intelligence. Moreover, progress in AI research will provide more solutions to the radiologists for predicting cancers. In the mere future, artificial intelligence will transcend as a prominent tool in clinical research.

## Figures and Tables

**Table 1:** Recently proposed algorithms for early diagnosis and classification of lung cancer.

S. No	Paper	Year	Input images	Dataset	Purpose	Classifier	Results
1	ALzubi et al. [28]	2019	Thoracic surgery dataset	1200	Lung cancer diagnosis	Weight Optimized NN with Maximum Likelihood Boosting classification	Feature selection rate - 90%
2	Pandiangan et al. [29]	2019	X-ray images	40	Lung cancer detection	ANN	Accuracy - 99%
3	Nasser et al. [30]	2019	Lung cancer dataset	NA	Lung cancer detection	Feed forward back propagation neural network	Accuracy - 96.67%
4	Roy et al. [31]	2019	Lung CT images	100	Lung cancer detection	SVM and Random forest algorithm	Efficacy - 94.5% Sensitivity - 74.2% Specificity - 77.6%
5	Bhalerao et al. [32]	2019	Lung CT images	90	Lung cancer detection	Maxpooling and ReLU algorithm	Accuracy – 94.34% Sensitivity – 91.755 Specificity – 95.7% Precision – 91.75%
6	Senthil et al. [26]	2018	Lung cancer image dataset	NA	Early detection of lung cancer	Partial swarm optimization	Accuracy - 97.8% Sensitivity - 94.8%
7	Perumal et al. [33]	2018	Lung CT images	100	Lung cancer detection and classification	Artificial bee colony optimization	Sensitivity - 92% True positivity rate - 92% False error rate - 7.6%
8	Xin Li et al. [34]	2018	Chest CT images	NA	Stage 1 diagnosis	CNN	Sensitivity - 96.4% Specificity - 95.6%
9	Wang et al. [25]	2018	Histopathology images	539	Discovery of tumor shape and boundary	CNN	Accuracy - 89.8%
10	Coudray et al. [27]	2017	Histopathology images	1175	Classification and mutation predication in NSCLC	Inception v3	Sensitivity - 97% Specificity - 97%

NN – Neural Network, ANN –Artificial Neural Network, SVM – Support Vector Machine, CNN – Convolution Neural Network, NSCLC – Non Small Cell Lung Cancer.

**Table 2:** Recently proposed algorithms for early diagnosis and classification of breast cancer.

S. No	Paper	Year	Input Data type	Dataset	Purpose	Classifier	Results
1	Batra et al. [48]	2020	Mammograms	161	Breast cancer detection	Max pooling	Accuracy (Tensorflow) - 87.98% Accuracy (Matlab) - 84.02%
2	Ali et al. [49]	2020	Mammograms	50	Breast cancer classification	Tetrolet transform based k- means classifier	Accuracy -92% Sensitivity - 88% Specificity - 96%
3	Kim et al. [50]	2020	Mammograms	17230	Detection of breast cancer	CNN	Accuracy - 95.9%
4	Wadkar et al. [51]	2019	Mammograms	5000	Breast cancer detection	ANN and SVM	Accuracy (artificial neural network) - 97% Accuracy (Support Vector Machine) - 91%
5	Alejandro et al. [52]	2019	Mammograms	240	Detection and classification of breast cancer	CNN	Accuracy - 89% Sensitivity - 86% Specificity - 79%
6	Alickovic et al. [53]	2019	Breast cancer dataset	699	Detection and classification of breast cancer	Perceptron neural network	Accuracy - 99.27%
7	Rodriguez-Ruiz et al. [54]	2019	Mammograms and breast tomosynthesis	9000	Detection of calcifications and soft lesions	Features classifier	Accuracy - 84%
8	Watanabe et al. [55]	2019	Breast cancer dataset	317	Breast cancer detection	Artificial intelligence-based computer-aided detection	Accuracy - 90%
9	Wang et al. [56]	2019	Mammograms	400	Breast cancer detection	Unsupervised extreme learning machine classifier	Accuracy of Single feature model - 76.25% Double feature model - 80.75% Multi feature model - 84.5%
10	Huang et al. [57]	2017	Breast cancer dataset	102993	Breast cancer prediction	SVM	Accuracy - 99.41%

CNN – Convolution Neural Network, ANN –Artificial Neural Network, SVM – Support Vector Machine.

**Table 3:** Recently proposed algorithms for early diagnosis and classification of gastric cancer.

S. No	Paper	Year	Input Data type	Dataset	Purpose	Classifier	Results
1	Aslam et al. [69]	2020	Saliva	220	Classification of gastric cancer into early and advanced stage	SVM	Accuracy - 97.18% Sensitivity - 96.88% Specificity - 97.44%
2	Li et al. [70]	2019	Endoscopic images	2429	Early diagnosis of gastric cancer	Inception v3	Accuracy - 90.91% Sensitivity - 91.18% Specificity - 90.64%
3	Guimarães et al. [71]	2019	OGDE images	200	Detection of gastric precancerous condition	CNN	Accuracy - 93%
4	Wang et al. [72]	2019	Gastroscopy images	104864	Screening of gastric cancer	CNN and SVM	Accuracy - 92.10%
5	Gao et al. [73]	2019	tomography images	1371	Detection of metastatic lymph nodes for gastric cancer classification	Faster region based CNN	Accuracy 95.45%
6	Leon et al. [74]	2019	Histopathological images	40	Detection of gastric cancer	CNN	Accuracy - 89.72%
7	Cho et al. [75]	2019	Endoscopic images	5017	Detection of gastric neoplasms	Inception Resnet v2 model	Accuracy - 84.6%
8	Wu et al. [63]	2018	OGDE images	24549	Early detection of gastric cancer	Deep CNN	Accuracy - 92.5% Sensitivity -94% Specificity - 91%
9	Sakai et al. [76]	2018	Endoscopic images	926	Automatic detection of gastric cancer	Transferring CNN	Accuracy - 82.8%
10	Zhu et al. [67]	2018	Endoscopic images	790	Prediction of invasion depth for endoscopic resection	CNN - computer aided detection system	Accuracy - 89.66% Sensitivity - 76.47% Specificity - 95.56%

OGDE images - Oesophagogastroduodenoscopic images, SVM – Support Vector Machine, CNN - Convolution Neural Network.
